# In Vitro Characterization of the Two-Stage Non-Classical Reassembly Pathway of S-Layers

**DOI:** 10.3390/ijms18020400

**Published:** 2017-02-14

**Authors:** Andreas Breitwieser, Jagoba Iturri, Jose-Luis Toca-Herrera, Uwe B. Sleytr, Dietmar Pum

**Affiliations:** Department of Nanobiotechnology, Institute for Biophysics, University of Natural Resources and Life Sciences Vienna, Muthgasse 11, Vienna 1190, Austria; andreas.breitwieser@boku.ac.at (A.B.); jagoba.iturri@boku.ac.at (J.I.); jose.toca-herrera@boku.ac.at (J.-L.T.-H.); uwe.sleytr@boku.ac.at (U.B.S.)

**Keywords:** S-layer proteins, two-step crystallization, self-assembly kinetics, non-classical crystal growth, Ca^2+^ binding

## Abstract

The recombinant bacterial surface layer (S-layer) protein rSbpA of *Lysinibacillus sphaericus* CCM 2177 is an ideal model system to study non-classical nucleation and growth of protein crystals at surfaces since the recrystallization process may be separated into two distinct steps: (i) adsorption of S-layer protein monomers on silicon surfaces is completed within 5 min and the amount of bound S-layer protein sufficient for the subsequent formation of a closed crystalline monolayer; (ii) the recrystallization process is triggered—after washing away the unbound S-layer protein—by the addition of a CaCl_2_ containing buffer solution, and completed after approximately 2 h. The entire self-assembly process including the formation of amorphous clusters, the subsequent transformation into crystalline monomolecular arrays, and finally crystal growth into extended lattices was investigated by quartz crystal microbalance with dissipation (QCM-D) and atomic force microscopy (AFM). Moreover, contact angle measurements showed that the surface properties of S-layers change from hydrophilic to hydrophobic as the crystallization proceeds. This two-step approach is new in basic and application driven S-layer research and, most likely, will have advantages for functionalizing surfaces (e.g., by spray-coating) with tailor-made biological sensing layers.

## 1. Introduction

Bacterial surface layer (S-layer) proteins have attracted much attention for more than four decades since their unique self-assembly properties allowed for specifically functionalized solid supports, such as silicon wafers or gold surfaces, with monomolecular crystalline lattices (termed S-layers or S-layer lattices) exposing chemical groups and biologically active domains in ordered dense packing [1,2,3,4]. In particular, the introduction of genetically modified S-layer-fusion proteins with incorporated custom-made functional domains, such as streptavidin for binding biotinylated molecules or nanoparticles, opened a new horizon for a broad range of applications in the life and material sciences [5]. However, these developments were only made possible based on extensive basic research concerning the structure, chemistry, genetics, morphogenesis and function of S-layer proteins (see the Table “Selected milestones in basic and applied S-layer research” [1]).

S-layer lattices represent the outermost cell envelope component in a broad range of prokaryotes (bacteria and archaea), and are one of the most abundant biopolymers on earth [1,2,5,6,7,8]. S-layers are isoporous protein or glycoprotein mesh works with unit cell sizes in the range of 3 to 30 nm, thicknesses of 5 to 10 nm (up to 70 nm in archaea), and pore sizes of 2 to 8 nm. Although no general biological function has been found so far, many of the specific functions assigned to S-layers depend on the complete covering of the bacterial cell and the physicochemical repetitive uniformity down to the sub-nanometer scale. 

Studies on the in vivo morphogenesis of S-layers demonstrated that, in order to maintain highly ordered closed monomolecular arrays on a growing cell surface, high rates of subunits must be synthesized, translocated to the cell surface and incorporated into the existing S-layer lattice [9]. Studies of *Bacillacaea* have demonstrated that a pool of S-layer subunits may be present in the peptidoglycan containing the cell wall matrix [6,10]. Within the S-layer pool, crystallization has to be avoided so that transport of the S-layer monomers upon secretion and fast incorporation into the outermost S-layer protein lattice is guaranteed.

Moreover, S-layer lattice formation on the surface of bacterial cells laid a foundation for studying the in vitro self-assembly properties of S-layer proteins in solution, at solid supports, lipid membranes, and at the air–water interface, and for utilizing them for biotechnological and biomedical applications [1,2,3,11]. In vitro self-assembly studies in solutions showed that self-assembly products (flat sheets, cylinders) are formed during dialysis of the disrupting agent against selected buffer solutions (ionic strength and pH) [2]. Kinetic measurements at different temperatures indicated that the assembly process is entropy driven with a rapid initial phase, at which oligomeric precursor patches acting as nucleation sites are formed, and a consecutive slow phase of crystal growth [12,13].

The first reassembly experiments with S-layer proteins at interfaces were carried out with the help of carbon coated transmission electron microscope (TEM) support grids inspected at certain time intervals in a TEM [14,15,16]. A major breakthrough in the characterization of S-layer lattice formation was facilitated by the introduction of atomic force microscopy (AFM; also often referred to as scanning force microscopy (SFM)) since then it was not only possible to image S-layer lattices on technologically relevant substrates such as silicon wafers or glass but—in particular—to investigate the interactions and assembly dynamics in real time [17,18,19,20,21,22,23]. In a seminal work, De Yoreo and co-workers were able to elucidate the non-classical pathway of crystal growth of the S-layer protein SbpA from *Lysinibacillus sphaericus* ATCC 4525 (ATCC; American type Culture Collection) on supported lipid bilayers of 1-palmitoyl-2-oleoyl-sn-glycero-3 phosphocholine (POPC) deposited on mica substrates [21]. The skillful use of state-of-the-art in situ AFM as required for working with very low protein concentrations (16 μg/mL versus 50–200 μg/mL in previous work) was key in addition to the development of a descriptive quantitative model. The results revealed a multi-stage assembly pathway consisting of four distinct processes: (i) adsorption of extended protein monomers onto the supported lipid bilayer; (ii) condensation into amorphous clusters; (iii) rearrangement and folding into crystalline arrays of tetramers; and (iv) growth by new tetramer formation at edge sites of the crystalline clusters [21,24]. Moreover, contrary to the classical assumption of S-layer crystal growth where crystals are considered to grow by the addition of monomeric units, oligomeric fragments, or even already formed entire morphological units, the emergence of order in the amorphous S-layer clusters was caused by a conformational transformation of the S-layer protein from its monomeric to the oligomeric form [21,24]. In this context, examination of the role of the substrate (without an attached lipid layer) demonstrated that, on bare mica, a kinetic trap associated with conformational differences between a long lived transient state and a final stable state plays an important role [22]. Both ordered tetrameric states emerged from clusters of the monomers but developed along two different pathways as figuratively expressed by the concept of a folding funnel [25]. While the final stable conformation was directly obtained, the trapped transient state changed only over time to its final low-energy state.

**Figure 1:**
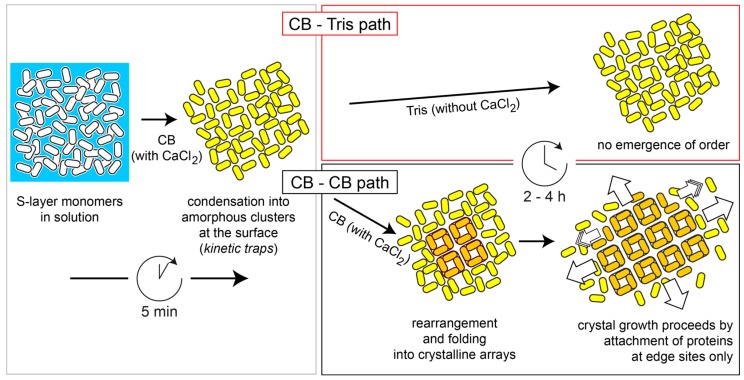
Schematic drawing of the two steps in the non-classical recrystallization process of the rSbpA S-layer protein. Adsorption of extended S-layer protein monomers in crystallization buffer (CB; containing CaCl_2_) from solution is completed within 5 to 10 min, leading to an irregular but complete coverage of the surface. At the same time, amorphous clusters of partially folded proteins are formed, which eventually transform within 2–4 h into crystalline arrays (in orange color) when crystallization buffer is added (CB–CB path). In the course of this step, the S-layer proteins undergo a refolding step into a more compact tetrameric form (in orange color). Crystal growth proceeds by attachment (and refolding) of new proteins at the edge sites of the growing lattice. In contrast, no crystalline lattice is formed (even not overnight) when only Tris—and no crystallization buffer is added (CB–Tris path).

The aim of this study focused on the separation of S-layer lattice formation into two steps, namely on (i) a short incubation of the substrate with SbpA S-layer protein leading to a layer of adsorbed protein followed by removal of excess material; and (ii) transition of the adsorbed amorphous clusters into a crystalline array exhibiting square (p4) lattice symmetry by addition of crystallization buffer (CB) containing CaCl_2_ but no further S-layer protein (Figure 1). The separation into the two consecutive but independent steps is new and allows the perfect control of the experimental parameters (e.g., with regular protein concentration, buffer composition, time evolution, etc.) for the generation of extended coherent crystalline S-layer lattices.

## 2. Results

The S-layer protein SbpA of *Lysinibacillus sphaericus* CCM 2177 is a perfect model protein to study crystallization pathways since the recrystallization process can only be initialized by the addition of a Ca^2+^ containing buffer [15,16,21,22,24,26,27,28]. A detailed discussion of the specific role of Ca^2+^-ions (and other divalent cations) associated with conformational changes within a protein and a more generic role mediating interactions between proteins (and supports) may be found in references [27,28,29]. The reassembly is entropy-driven and a fascinating example of matrix assembly following a multistage, non-classical pathway in which the process of S-layer protein folding is directly linked with assembly into extended clusters [13]. In a theoretical approach, the formation of an S-layer lattice with square (p4) lattice symmetry (as for SbpA) was investigated by assuming both non-specific attractive and specific directional bonds for the monomers [30,31,32]. The results were in very good agreement with the experimental findings by showing, for example, that liquid-like clustering may precede crystallization.

### 2.1. Recombinant S-Layer Protein rSbpA

The S-layer protein SbpA from *Lysinibacillus sphaericus* CCM2177 (equivalent to ATCC 4525, [33]) was used because SbpA is one of the most studied S-layer protein model systems. However, we focused on the recombinant rSbpA S-layer protein (instead of the wild type strain) because only the usage of the recombinant protein, identical structure and length to the wild type protein, allowed reproducible reassembly experiments, in particular when the influence of Ca^2+^ in coating and recrystallization experiments was investigated. Particularly for the latter experiments with Ca^2+^, reproducible results could not be achieved with the wild type strain. This was because different production batches of the wild type SbpA protein also recrystallized into a closed crystalline monolayer even when no Ca^2+^-ions (with buffer) were added (Figure S1). Previous studies indicated that impurities or possibly traces of the secondary cell wall polymer (SCWP), the naturally occurring substrate within the cell wall, obscures the necessity of Ca^2+^-ions. However, as a consequence of the recombinant production, SCWP cannot be present in rSbpA S-layer protein solutions and thus allows unambiguous results. Identical to the wild type strain, rSbpA reassembles into monolayer arrays with square (p4) lattice symmetry and a lattice spacing of a = 13.1 nm [34].

### 2.2. Quartz Crystal Microbalance with Dissipation (QCM-D) Studies

Quartz crystal microbalance with dissipation (QCM-D) allowed a detailed analysis and quantification of both adsorption and the subsequent recrystallization process in real time. In QCM-D measurements, the change in frequency describes the amount of adsorbed mass per unit area while dissipation its elastic/viscoelastic behavior. Initial experiments with QCM-D (Figure S2) showed that, after 5 min of incubation with a monomeric recombinant rSbpA S-layer protein solution (50 µg/mL in crystallization buffer), no further binding of S-layer protein at the silicon wafer took place even with excess S-layer protein in the solution.

**Figure 2:**
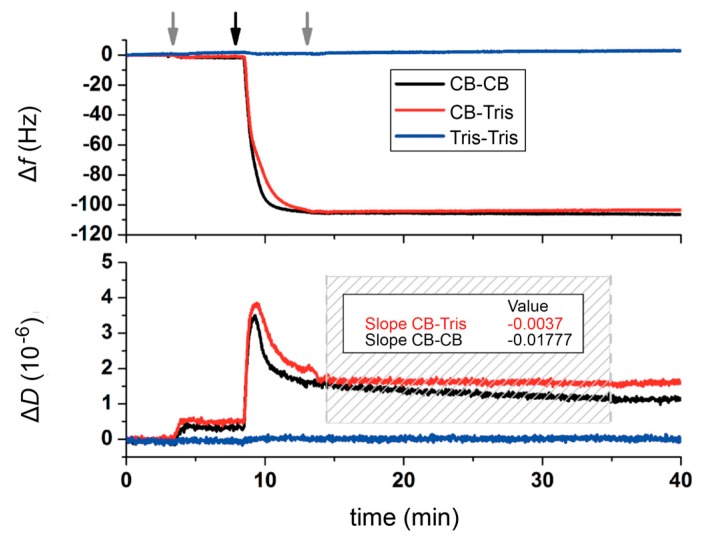
Real-time monitoring of rSbpA protein adsorption on hydrophobic silicon quartz crystal microbalance with dissipation (QCM-D) chips as recorded for frequency (top) and dissipation (bottom). Grey arrows indicate buffer injection, while the black arrow refers to injection of rSbpA monomeric solution. A dashed box highlights the post-rinse region employed to fit a linear dissipation decay. Respective slopes of the fitting curves are shown in the box.

Figure 2 shows the change in frequency and dissipation for different buffer combinations (with and without Ca^2+^-ions) for the adsorption and lattice formation of rSbpA on hydrophobic silicon wafers. As observed, rSbpA injected into the crystallization buffer interacted very rapidly with the substrate and induced a frequency decrease (∆*f*) down to values around −100 Hz within the first 2–3 min, corresponding to an increase in deposited mass approximately of 1.8 µg/cm^2^. Frequency values remained constant then for the rest of the experiment—despite a thorough rinsing performed in the corresponding buffer solution (right grey arrow in Figure 2). An unambiguous differentiation between the influence of either the addition of crystallization buffer or, alternatively, the rinsing with pure Tris buffer (identically to crystallization buffer but without Ca^2+^; 5 mM Tris; pH 9.0) was only possible by careful evaluation of variations in dissipation (∆*D*). For comparison, the change in ∆*f* is not suitable for such a minute investigation. However, after rSbpA injection, the characteristic peak in ∆*D* is followed by two distinctive different post-rinse behaviors characterized by either no (CB-Tris) or a gradual (CB-CB) decay (Figure 2). The respective slopes indicate this difference (at a ratio of 1 to 5) within the dashed area shown in Figure 2. Since the total shift in ∆*D* (of around 0.5) for the CB–CB system represents a decrease of 30% from values already representing high compactness (as shown by the low starting value of ∆*D* = 1.5), the ongoing structural rearrangement is highly relevant for such a thin film.

**Figure 3:**
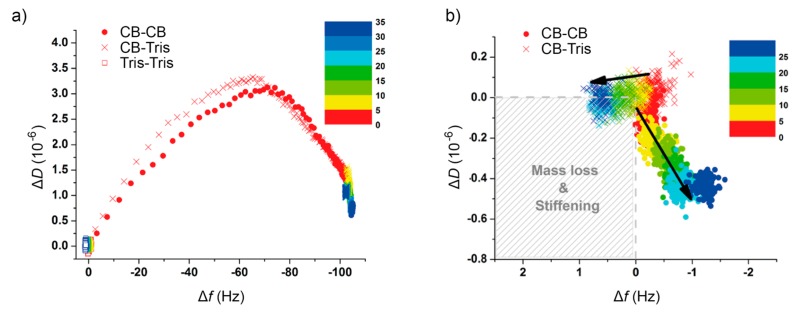
(**a**) Df plot showing the full process (adsorption + rinse) of rSbpA on hydrophobic silicon chips after short (5 min) incubation in different buffers; (**b**) magnification of the post-rinse evolution of the protein films upon rinse with either Tris buffer or crystallization buffer (CB; containing Ca^2+^-ions). The respective color scales indicate the time elapsed since the corresponding injections (in min).

In this regard, the use of Tris buffer seemed to prevent the reorganization of the adsorbed protein film, contrary to what is observed in the presence of Ca^2+^-ions, which evolves towards a structure of higher rigidity within the following 20–30 min. A different approach to explore such a behavior is depicted in Figure 3a, where, in a color-coded way, the rSbpA–substrate interaction process may be analyzed by displaying the changes in ∆*f* and ∆*D* simultaneously over time. The almost identical behavior in ∆*f* during the first 5 min for both systems in the presence of Ca^2+^, including the aforementioned transition peak at −65 Hz, differs drastically after rinsing with different buffers. A closer look at such post-rinsing behavior (Figure 3b) clearly indicates the continuing dense packing experienced for rSbpA in a crystallization buffer as compared to almost no variation recorded in the Tris buffer (see Figure 1 for a schematic drawing). The slight mass uptake measured for the system under reorganization may be interpreted as further proof of the transformation of the amorphous clusters into an array of compact tetramers, and, in this way, the refolding of the monomer upon crystal formation.

In turn, the injection of rSbpA in the presence of Ca^2+^-free Tris buffer should be considered apart from the description given above. As shown in Figure 2 and Figure 3, the absence of Ca^2+^ in the bulk solution does not only dramatically affect the reorganization of the S-layer clusters but also the efficiency in the adsorption of the S-layer proteins on the solid support. Indeed, in the absence of Ca^2+^, the interaction of rSbpA with the hydrophobic surface simply does not take place. As mentioned before, the use of recombinant S-layer protein rSbpA enabled more controlled coating experiments than with the wild type SbpA strain (Figure S1).

We consider QCM-D as a complementary method to direct imaging approaches such as in situ AFM described below. QCM-D provides quantitative results (adsorbed mass and its viscoelastic properties and, in this way, emerging crystalline order) and a view of a much larger area (typically 1 cm^2^ (=10^8^ μm^2^) for a QCM-D chip) compared to AFM (typically, 1 μm^2^ or less). Moreover, QCM-D usually provides a much better time resolution than AFM: for example, within the first 5 min, around 150 data points (roughly every 1.5 s) were taken while, at the same time, only a few AFM images would have been captured. Moreover, as shown in Figure 3b in the next 30 min, the minute trend towards mass loss and stiffening for the CB-Tris path while, in comparison, away from those for the CB–CB path would probably have been challenging for AFM. However, aside from the straightforward approach of in situ AFM for studying crystal growth, there is also the possibility that the AFM tip might interact with individual S-layer proteins, in particular at the moment when they are binding to a crystalline patch, and thus influence the results.

### 2.3. Atomic Force Microscopy (AFM)

Based upon these findings, the starting point for AFM investigations were silicon chips incubated with rSbpA (50–200 µg/mL crystallization buffer) for 5 or 10 min, respectively. Unbound S-layer protein was washed away and lattice formation was allowed to take place in crystallization buffer overnight. In contrast to incubation in Tris or Milli-Q water (Merck Millipore, Darmstadt, Germany), an S-layer lattice with square (p4) lattice symmetry could be clearly seen confirming the capability of the adsorbed S-layer protein to form a closed compact monolayer. Lower concentration of applied rSbpA (10 and 20 µg/mL crystallization buffer; 10 min) or shorter initial incubation times (1 and 2 min) did not lead to closed crystalline lattices after washing the substrates and incubation in crystallization buffer overnight. It was assumed that, in this situation, the incubation time was too short and the concentration of S-layer proteins too low for a sufficient amount of protein adsorbed on the silicon surface. The most representative experimental set up was the incubation of rSbpA at a concentration of 50 µg/mL crystallization buffer for 5 min. After washing away unbound S-layer protein, the crystalline lattice structure could clearly be seen by AFM when the coated substrates were further incubated in a crystallization buffer (average domain size 1–2 μm). When the additional incubation was carried out in Tris buffer (without CaCl_2_) or Milli-Q water, the S-layer lattice structure could not be visualized.

These findings were also supported by the observation that the crystallization process or the transformation of the adsorbed amorphous into the crystalline state was terminated by crosslinking the S-layer protein clusters with glutardialdehyde (Figure S3). After 5 min incubation with recombinant S-layer protein (50 µg/mL), unbound protein was washed away and the remaining S-layer protein (on the silicon wafer) immediately cross-linked (Figure S3a). In this situation, a protein lattice structure could not be observed. When the crosslinking step was performed after incubation in crystallization buffer, a closed crystalline monolayer could be seen, proving, on the one hand, the transformation of the amorphous into the crystalline state and, on the other hand, that the crosslinking step does not interfere with the visualization of the crystalline structure in AFM investigations (Figure S3b).

**Figure 4:**
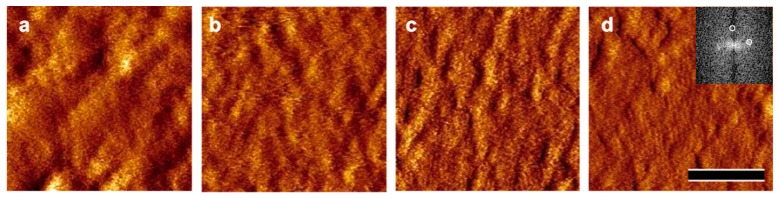
Atomic force microscopy (AFM) pictures of rSbpA recrystallized on a silicon wafer exploiting the two-stage process. The recombinant S-Layer protein rSbpA was incubated for 10 min (50 µg/mL crystallization buffer) and, after a washing step, incubation was prolonged. The change from the adsorbed state into the crystalline state was monitored by taking images after 10 min (**a**); 60 min (**b**); 120 min (**c**); and 140 min (**d**), respectively. The square (p4) lattice structure could be visualized after 120 min incubation in crystallization buffer (bar, 200 nm). Inset shows the first order peaks of the reciprocal lattice in the Fourier spectrum corresponding to a lattice spacing of 13 nm in real space.

Additionally, in situ AFM investigations were performed in order to determine the time required to obtain the crystalline lattice after the adsorption of S-layer protein on the surface. Again, silicon wafers were incubated with rSbpA (50 µg/mL crystallization buffer for 5 min), washed, and subsequently inspected by AFM. Crystallization buffer was dropped onto the silicon wafer and AFM images were taken at certain time intervals. At early incubation times (after 10, 20 and 60 min), it was challenging to visualize the crystalline lattice, but, after 120 and 140 min, the closed crystalline monolayer exhibiting the square (p4) lattice structure could be unambiguously detected (Figure 4). It was concluded that the amount of protein adsorbed was sufficient to form a closed monolayer, and, although the crystalline layer emerged subsequently, the transformation of the amorphous to the crystalline state and, in this way, the increase in rigidness requires a certain amount of time and the presence of Ca^2+^-ions (e.g., CaCl_2_). The difficulty in obtaining in situ AFM images (with sufficient quality) at early stages in the assembly process is explained by the extreme softness of the protein layer at this time. This is particularly true for the very first minutes when the S-layer proteins are only loosely attached towards the support and prone to movement and rotations into all possible directions by the AFM tip. Nevertheless, in the course of the subsequent reorientation against the surface and, in this way, stronger binding, ∆*D* decreases and subsequently in situ AFM becomes able to show emerging order in the protein layer.

For the sake of completeness, when hydrophilic substrates (plasma treated silicon wafers) were used, adsorption and crystallization required a longer time (overnight) and, following the two-step approach, the crystalline domains appeared only as small individual patches (average diameter 3–4 μm) (data not shown).

### 2.4. Contact Angle Measurements

**Figure 5:**
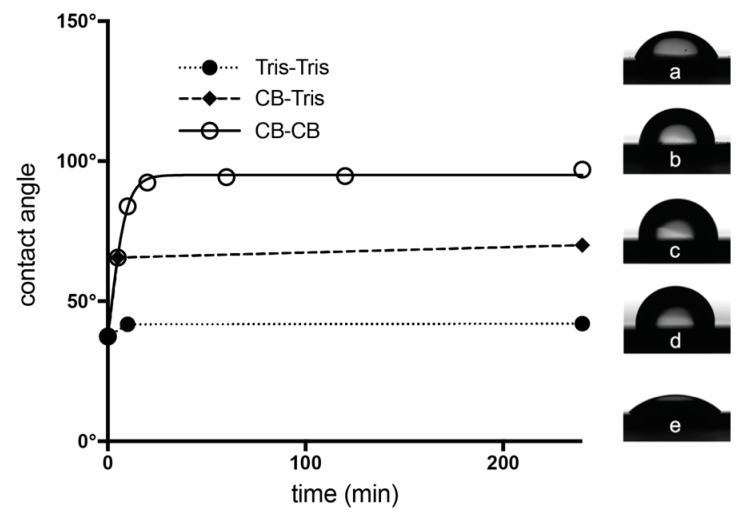
The contact angles of SbpA coated Si wafers are shown in a time-dependent manner. The contact angle of blank silicon was 37° (t = 0 min). After incubation with the S-layer protein and washing away unbound protein, recrystallization was allowed to take place in recrystallization buffer for 5 min (**a**) (insets demonstrate the CB–CB path), 10 min (**b**), 20 min (**c**), 60 min, 120 min and 240 min (**d**), respectively. The contact angles were measured after air-drying the coated substrates. Inset (**e**) shows the contact angle of the S-layer coated silicon wafer without CaCl_2_ in the buffer (Tris–Tris path; after 10 min).

Additional information about the time evolution of the change in crystallinity of the S-layer was obtained with contact angle measurements. An increase in hydrophobicity of S-layer coated silicon wafers was observed during the advancing recrystallization time (Figure 5). The blank silicon wafers (contact angle of 37° ± 1.8°) were incubated with rSbpA in crystallization buffer (50 µg/mL) for 5 min. After washing away the unbound S-layer protein, further incubation was performed in the crystallization buffer. After 5 min of S-layer reassembly, the contact angle had changed to 66° ± 0.7°, indicating a much higher hydrophobicity. After 10 min, the contact angle was in the range of 84° ± 0.3° (without CaCl_2_ in the buffer, the contact angle was 42° ± 0.4°, and did not change any longer). After 20 min, the contact angle values had already reached a plateau since longer recrystallization times led only to a minor increase in hydrophobicity. Overnight incubation led to a contact angle of 98° ± 2.7° for S-layer coated silicon wafers. These findings were in good agreement with QCM-D measurements, where also, after 5 min, no further increase of adsorbed S-layer protein was observed, but the rigidness still increased, and, by AFM, where the lattice became visible then too. Thus, these findings may be considered as further evidence for the change in the crystalline state (from adsorbed to crystalline) and also in the rigidity of the S-layer. 

## 3. Discussion

In this work, we introduce a new protocol to form 2D crystalline monolayers of SbpA with unprecedented control over the induction of crystallinity. QCM-D and AFM have demonstrated that the adsorption of S-layer proteins and their transition into the crystalline state follows a multi-stage non-classical pathway. Adsorption was completed within 5 min and a closed monomolecular crystalline S-layer obtained after a prolonged incubation time in a buffer containing CaCl_2_. The initially adsorbed amount of protein was sufficient for covering the entire surface area. This could be visualized by AFM with real-time monitoring or by stopping the recrystallization process by crosslinking and conserving the actual state of the crystallinity. In addition, QCM-D measurements supported these findings where the increasing rigidity could only be observed when—after the adsorption and washing step—the incubation was prolonged in CaCl_2_ containing buffer. The results of the CB–CB path, which may be considered as equivalent to the classical “one-step” approach, fit perfectly well to the existing literature (for reviews, see [1,2,35,36]). In the future, we would like to anticipate the generalizability of the two-step approach for all those S-layer proteins where the induction of crystallinity may be triggered by a specific buffer composition (e.g., the presence of Ca^2+^ for SbpA [17,27] or slp-B53 [28]).

Moreover, contact angle measurements showed that the surface properties of S-layers change from hydrophilic to hydrophobic as the crystallization proceeds. This observation supports the phenomenon of the unique anti-fouling properties of crystalline S-layers—also as a benefit for the bacterial cell [37].

The growth mechanism in a non-classical pathway is entirely different to the classical assumption where crystal growth is described by the addition of completely folded monomeric units or oligomeric fragments, or even entire morphological unit cells [35,36,38,39,40,41,42]. We assume that extended monomers are attached to the surface and are first amorphous and in the following microcrystalline clusters, from which crystalline order emerges, are formed. However, it is currently under discussion how many monomeric units constitute a nucleation site with sufficient internal order to initiate lattice growth along the formed domain [2,41]. Concentration-dependent dynamic light scattering (DLS) measurements provided evidence of a “critical concentration” of association in the range of 12 to 16 monomers for the S-layer protein of *B. stearothermophilus* NRS 1536/3a (showing square (p4) lattice symmetry) [12], and, accordingly, in situ AFM yielded eight to 60 monomers for SbpA [21]. In addition, theoretical work has also supplied very valuable information about the event leading to the spontaneous formation of nucleation sites [30,31,43]. Moreover, the presence of amorphous clusters—as associated with the non-classical pathway—may also be responsible for a self-purifying effect in the course of crystal growth [41]. This is particularly true for reassembly in solution where the S-layer monomer concentration is up to twenty times higher compared to the reassembly on solid surfaces as discussed here. The question of self-purification is particularly interesting when discussing the heterologous reattachment of two different S-layer proteins [4]. *Thermoanaerobacter thermosaccarolyticum* reassembles into regular arrays with square (p4) lattice symmetry while *Thermoanaerobacter thermohydrosulfurium* reassembles into hexagonal (p6) lattice symmetry, respectively. When a mixture of both S-layer protein species was supplied, small but distinct arrays of both lattice types were formed individually. It has to be stressed that, for each of the two S-layer proteins species, the other one was an impurity. Nevertheless, individual crystalline domains were formed, supporting the concept of self-purification pathways as induced by amorphous cluster assimilation [4,41].

Finally, it has to be reported that the results of this work also allowed for functionalized surfaces by spray-coating and subsequent incubation in a crystallization buffer. Perfectly ordered S-layer protein monolayers were obtained with a minimum of material usage. We would like to anticipate that this new approach is highly attractive for all applications where a continuous coating with a biological sensing layer based on tailor-made S-layer fusion proteins, such as affinity matrices in biosensor developments, plays a key role in the fabrication process [1,5,8].

## 4. Materials and Methods

### 4.1. Production of Monomeric S-Layer Protein Solutions

The recombinant S-layer protein rSbpA from *Lysinibacillus sphaericus* CCM 2177 was constructed, cloned, and heterologously expressed in *Escherichia coli* HMS174 (DE3) as described in references [44,45]. The recombinant S-layer protein was over-expressed in *E. coli* and accumulated in inclusion body like structures, which were stored after a downstream processing including a homogenization step at −20 °C [26,46]. Subsequently, the protein was extracted with 5 M guanidine hydrochloride (GHCL, Gerbu Nr. 1057, GERBU, Heidelberg, Germany) and applied to gel chromatography as described previously [46]. The pooled fraction containing the S-layer protein was dialysed (membrane type 27 Nr. 08400 cut-off 12–16 kD, pore size 25A, Biomol, Hamburg, Germany) twice against 3 L Milli-Q (Merck Millipore, Darmstadt, Germany) water containing 2 mM ethylenediaminetetraacetic acid (EDTA, Gerbu Nr. 1034, GERBU, Heidelberg, Germany) at room temperature (22 °C) followed by a dialysis step at 4 °C overnight. After the dialysis step, the protein solution was filtrated through a 0.2 µm syringe filter and used immediately. It has to be stressed that the monomeric protein solution might have contained oligomers too.

To determine the protein concentration of the obtained rSbpA monomeric protein solution, UV measurements were performed at 280 nm using a spectrometer (U-2900, Hitachi, Tokyo, Japan) and a quartz cuvette. The protein concentration was adjusted to 1 mg/mL using ice-cold Milli-Q water using the extinction coefficient for SbpA (extinction at 280 nm × 1.64 = concentration in mg/mL). To confirm the recrystallization properties onto solid surfaces, the obtained monomeric protein solution was controlled by means of AFM as described previously [18]. The protein solution was then stored at 4 °C for a maximum of 4 weeks. 

### 4.2. Quartz Crystal Microbalance with Dissipation (QCM-D) Monitoring

QCM-D experiments were performed in a Q-Sense E4 instrument (Q-Sense, Biolin Scientific AB, Västra Frölunda, Sweden). Prior to their use in the experiments, silicon dioxide coated quartz sensors (QSX 303, Q-Sense AB) were sonicated in 2% (*w*/*w*) sodium dodecyl sulfate (SDS) solution for 20 min and rinsed with ultrapure water and ethanol. The crystals were dried in a stream of nitrogen, subsequently treated with UV/Ozone for 30 min and left overnight under saturated atmosphere of 1H, 1H, 2H, 2H-perfluorodecyltrichlorosilane in chloroform in a vacuum chamber to ensure their hydrophobicity. Afterwards, silanized sensors were sonicated in ultrapure water and ethanol and finally mounted into the QCM-D chamber. Experiments were performed at 25 °C. Real-time variations of frequency (∆*f*) and dissipation (∆*D*) parameters were observed at several overtones (*n* = 3, 5, 7,..., 13) throughout the experiments. Injection of SbpA protein (50 µg/mL), all washing steps as well as the addition of the different buffers were performed by means of a peristaltic pump (Ismatec, Cole-Parmer, Wertheim, Germany) operating at a flow rate of 0.3 mL/min.

Additionally, ∆*D* versus ∆*f* plots were used in order to provide a more detailed view of the viscoelastic evolution of the S-layer array in terms of a mass unit (∆*m*) change. Each data point in a ∆*D*/∆*f* plot represents a dissipation/frequency-pair at a certain time. ∆*D*/∆*f* plots, as well as derived slopes, are characteristic for specific situations in the course of the reassembly processes and, in this way, allow a differentiation between them.

### 4.3. Recrystallization of S-Layer Proteins on Silicon Wafers

Silicon wafers (with a native silicon oxide layer) (Si-Mat, Kaufering, Germany) were rinsed with acetone, ethanol, Milli-Q water and carefully dried in a stream of nitrogen gas. Immediately prior to incubation, the monomeric rSbpA protein solution was diluted with crystallization buffer (5 mM Tris; 10 mM CaCl_2_, pH = 9.0) to a concentration of (typically) 100 µg/mL. In general, the support was immersed into the S-layer protein solution and incubation lasted overnight. The conversion of adsorbed protein into a crystalline monomeric structure was investigated by incubating the silicon wafers with S-layer protein solution at various concentrations (10, 20, 50, 100 and 200 µg/mL) for 5 and 10 min at room temperature (22 °C). Subsequently, unbound S-layer protein was removed in a washing step with crystallization buffer and Milli-Q water. Then, the coated wafers were incubated overnight at 4 °C in a crystallization buffer in order to allow the finishing of the crystallization process. For comparison, as blanks, wafers with adsorbed S-layers were also incubated in a 5 mM Tris buffer (pH = 9.0) in the absence of Ca^2+^ ions overnight. Alternatively, the crystallization process could be stopped after the S-layer incubation (5 and 10 min) by crosslinking the protein with glutardialdehyde (0.25% in 100 mM phosphate buffer, pH 7.2) for 20 min.

The crystallization process was also monitored by AFM. For this purpose, silicon wafers were incubated with rSbpA (30 µg/mL crystallization buffer) and, after several washing steps, transferred to the AFM. After deposition of a drop of crystallization buffer onto the wafer, the advancing crystallization could be observed. Images were taken after 10, 20, 60, 120 and 140 min.

### 4.4. Atomic Force Microscopy

Atomic force microscopy (AFM) was carried out with a Multimode AFM (Bruker AXS, Santa Barbara, CA, USA) equipped with a Nanoscope-V controller and an E-scanner (scan range up to 12 µm). Silicon-nitride probes (MSNL-10, Bruker AXS, Santa Barbara, CA, USA) with a nominal spring constant of 0.2 N/m were used in the experiments. The actual spring constants were determined by the thermal tuning method [47,48]. Samples were investigated in contact mode, which allowed faster scan rates as well as a more accurate monitoring and visualization of the coated substrates in comparison to the tapping mode. 

### 4.5. Contact Angle Measurements

The relationship of the crystalline state and hydrophobicity of the supporting silicon surfaces were investigated by determining the contact angles of S-layer coated silicon wafers after 5, 10, 20, 60, 120 and 240 min incubation times. S-layer solution was applied at a concentration of 50 µg/mL in crystallization buffer for 5 min. After a washing step, incubation time was prolonged in crystallization buffer for 5, 10, 20, 60, 120 and 240 min (CB–CB path). The CB-Tris path was monitored by further incubation in Tris for 10 min and 240 min. An uncoated wafer and a wafer where the S-layer protein was applied and, after a washing step, further incubated in Tris buffer but in the absence of CaCl_2_ for 10 min and 240 min (Tris-Tris path), were used as blank or for comparison, respectively. Contact angles were determined with a Kruess EasyDrop instrument (Kruess, Hamburg, Germany) using 8 µL Milli-Q water droplets. 

## Supplementary Materials

Supplementary materials can be found at www.mdpi.com/1422-0067/18/2/400/s1.
