# Supplementary material for: In Vitro Characterization of the Two-Stage Non-Classical Reassembly Pathway of S-Layers
Source: Int J Mol Sci. 2017 Feb 14;18(2):400. doi: 10.3390/ijms18020400 (PMC5343934; doi:10.3390/ijms18020400)
Supplement: Supplementary file 1 [file ijms-18-00400-s001.docx]

Supplementary Materials: In Vitro Characterization of the Two-Stage Non-Classical Reassembly Pathway of S-Layers

Andreas Breitwieser, Jagoba Iturri, Jose-Luis Toca-Herrera, Uwe B. Sleytr and Dietmar Pum


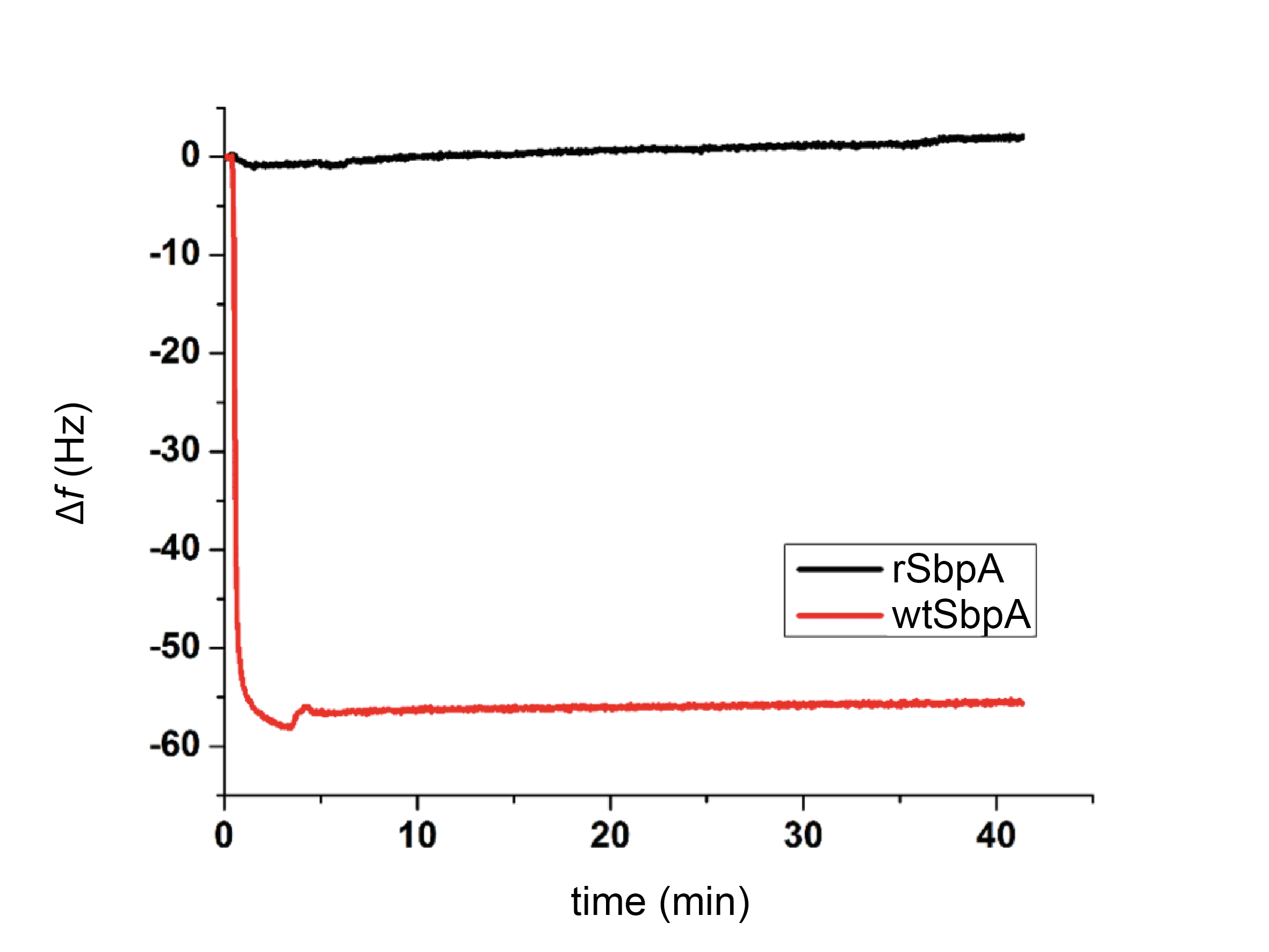


**Figure S1.** Frequency change monitored for the injection of either recombinant (rSbpA, in black) or wild type (wtSbpA, in red) S-layer proteins in Tris buffer.


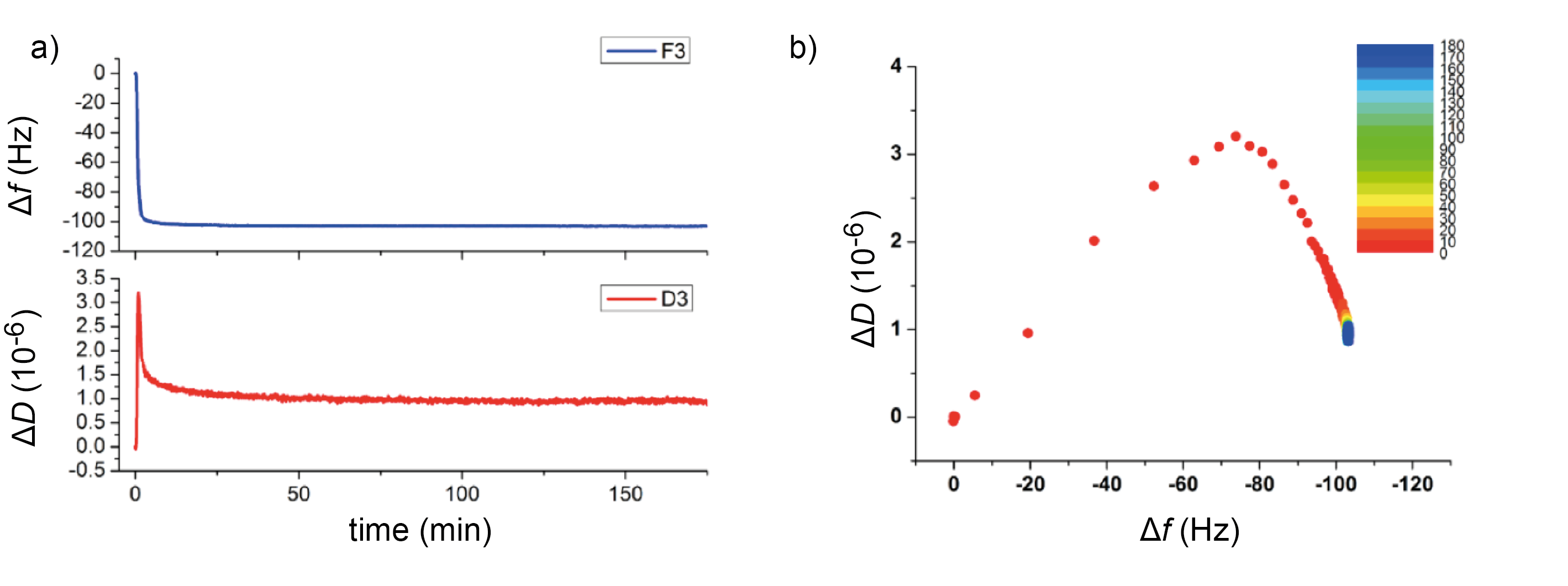


**Figure S2.** (**a**) Representative crystal microbalance with dissipation (QCM-D) plot for long-term (180 min) incubation of rSbpA on hydrophobic Silicon dioxide; (**b**) Df plot obtained from the time evolution of ∆*f* and ∆*D* in (**a**). The color scale refers to the time elapsed after rSbpA S-layer protein injection.


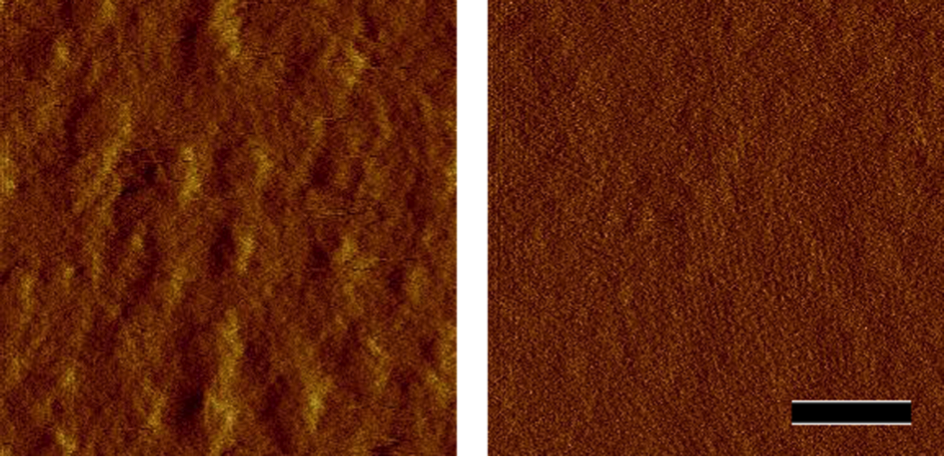


**Figure S3.** Atomic force microscopy (AFM) pictures of the recombinant S-Layer protein rSbpA recrystallized on a silicon wafer. rSbpA was incubated for 5 min (100 µg/mL crystallization buffer). A protein lattice structure could not be observed when a crosslinking step with glutardialdehyde was performed in due course (after a washing step) (**a**). But, when the crosslinking step was carried out after incubation in crystallization buffer (at least for 120 min) a closed crystalline monolayer was visible (**b**); (bar, 200 nm).
